# The effects of a commercially available botanical supplement on strength, body composition, power output, and hormonal profiles in resistance-trained males

**DOI:** 10.1186/1550-2783-7-34

**Published:** 2010-10-27

**Authors:** Chris Poole, Brandon Bushey, Cliffa Foster, Bill Campbell, Darryn Willoughby, Richard Kreider, Lem Taylor, Colin Wilborn

**Affiliations:** 1 Human Performance Lab, Department of Exercise and Sport Science, University of Mary Hardin-Baylor. Belton, Texas, 76513, USA; 2 Exercise and Performance Nutrition Lab, School of Physical Education and Exercise Science, The University of South Florida, USA; 3 Exercise and Biochemical Nutrition Laboratory, Department of Health, Human Performance & Recreation; Baylor University, Waco, TX 76798, USA; 4 Exercise and Sport Nutrition Laboratory, Department of Health and Kinesiology, Texas A&M University, College Station, TX 78743, USA

## Abstract

**Background:**

Fenugreek (*Trigonella foenum-graecum*) is a leguminous, annual plant originating in India and North Africa. In recent years Fenugreek has been touted as an ergogenic aid. The purpose of this study was to evaluate the effects of Fenugreek supplementation on strength and body composition.

**Methods:**

49 Resistance trained men were matched according to body weight and randomly assigned to ingest in a double blind manner capsules containing 500 mg of a placebo (N = 23, 20 ± 1.9 years, 178 ± 6.3 cm, 85 ± 12.7 kg, 17 ± 5.6 %BF) or Fenugreek (N = 26, 21 ± 2.8 years, 178 ± 6 cm, 90 ± 18.2 kg, 19.3 ± 8.4 %BF). Subjects participated in a supervised 4-day per week periodized resistance-training program split into two upper and two lower extremity workouts per week for a total of 8-weeks. At 0, 4, and 8-weeks, subjects underwent hydrodensiometery body composition, 1-RM strength, muscle endurance, and anaerobic capacity testing. Data were analyzed using repeated measures ANOVA and are presented as mean ± SD changes from baseline after 60-days.

**Results:**

No significant differences (p > 0.05) between groups were noted for training volume. Significant group × time interaction effects were observed among groups in changes in body fat (FEN: -2.3 ± 1.4%BF; PL: -0.39 ± 1.6 %BF, p < 0.001), leg press 1-RM (FEN: 84.6 ± 36.2 kg; PL: 48 ± 29.5 kg, p < 0.001), and bench press 1-RM (FEN: 9.1 ± 6.9 kg; PL: 4.3 ± 5.6 kg, p = 0.01). No significant interactions was observed among groups for Wingate power analysis (p = 0.95) or muscular endurance on bench press (p = 0.87) or leg press (p = 0.61). In addition, there were no changes among groups in any clinical safety data including lipid panel, liver function, kidney function, and/or CBC panel (p > 0.05).

**Conclusion:**

It is concluded that 500 mg of this proprietary Fenugreek extraction had a significant impact on both upper- and lower-body strength and body composition in comparison to placebo in a double blind controlled trial. These changes were obtained with no clinical side effects.

## Background

Fenugreek (*Trigonella foenum-graecum*) is a leguminous, annual plant originating in India and North Africa. It is an herbal product with many proposed health benefits found in the diets of various Middle Eastern countries and is now cultivated worldwide. The leaves and seeds of fenugreek are formulated to an extract or powder form for therapeutic application.

Fenugreek has been studied extensively in human and animal models. The effects of fenugreek supplementation on the regulation of insulin and hyperglycemia are well established. Defatted fractions of fenugreek seeds, high in fiber content and containing steroid saponins, lowered blood glucose and plasma glucagon concentrations after eight days of consumption in dogs [1]. Other investigations utilizing human participants have implemented fenugreek supplementation (daily doses of 1 to 25 g/day) to diabetic patients eliciting positive glucose regulation responses [2,3]. Another study [4] examined the acute and chronic outcomes of a soluble dietary fiber (SDF) prepared from fenugreek seeds administered to type 1 and type 2 diabetic rats. After an oral glucose cocktail, SDF significantly offset blood glucose elevation in non-diabetic and diabetic (type 1 and 2) rats at 75 and 30 minutes post-consumption respectively. Following a 28 day SDF supplementation period, type 2 diabetic rats experienced a significant reduction (19%) in blood glucose levels, initiating a 1.5 fold increase in hepatic glycogen stores. Other formulations of fenugreek, such as the combination of several oils (including fenugreek oil), have shown to decrease circulating glucose and enhance insulin sensitivity in diabetic and hypertensive rats [5]. The glucose transporting mechanisms observed in these studies are mediated though an insulin-signaling pathway [6]. Fenugreek seed extract acts in a similar fashion to that of insulin by promoting glucose uptake into cells through a dose-dependent manner [6]. Additional evidence has shown that fenugreek seeds aid in the release of insulin from pancreatic beta cells [7], thus allowing blood glucose levels to reduce by the transport and entrance of glucose into muscle cells.

Fenugreek has shown to be a useful remedy in combating abnormal cholesterol profiles in hyperlipidemic populations. A daily dose of fenugreek seed administered to rats (100 or 500 mg/kg) for eight weeks lowered LDL, VLDL triglyceride and total cholesterol and increased HDL when compared to a control group [8]. Fasting cholesterol and triglyceride levels were similar across groups when fed either a high-cholesterol diet with fenugreek extract or a standard diet [9], and post-prandial triglyceride levels were higher in rats on the standard diet [9] concluding that fenugreek reduces triglyceride levels in fasting and post-prandial states.

There is also evidence linking fenugreek to reduced hepatic cholesterol levels and elevated hepatic triglyceride lipase (HTGL) activity [10], the enzyme accountable for catabolizing chylomicrons and VLDL's to smaller remnant particles [11]. Mitigation of hepatic steatosis by reducing triglyceride accumulation in the liver [12] and prevention of ethanol-induced toxicity and apoptosis in liver cells [13] are other recent discoveries attributable to fenugreek. An aqueous herbal extract containing fenugreek lowered alanine aminotransferase (ALT), aspartate aminotransferase (AST), and glucose values, signifying a reduction in inflammation and a feasible protective agent against alloxan-induced oxidative stress and diabetes [14].

Animal studies have demonstrated that Fenugreek possesses ergogenic as well as anabolic properties. One inquiry reported that fenugreek (300 mg/kg) increased swimming time to exhaustion in rats after four weeks of supplementation [15], perhaps due to increased utilization of fatty acids during exercise. A trial performed on male rats found that after four weeks, Galactomannan supplementation (isolated from fenugreek seeds) was as effective in increasing weight of the levator ani muscle to that of testosterone treatment [16]. Likewise, a compound containing the steroidal sapogenin diosgenin, which is found in Fenugreek seeds, augmented overall weight and muscle growth in rats when compared to control subjects [17]. The anabolic properties of fenugreek observed in the mentioned animal studies have yet to be determined in humans. There is no research to date that has investigated the effects of fenugreek in humans on strength, anaerobic exercise performance, or hormonal changes in humans. Therefore, the purpose of this study was to determine the effects of a commercially available supplement containing *Trigonella foenum-graecum *on strength, body composition, power output, and hormonal profiles in resistance-trained males over the course of a structured resistance training program.

## Methods

### Experimental Approach to the Problem

The study was conducted as a double-blind, placebo controlled trial using parallel groups matched according to total body weight. The independent variable was the nutritional supplement *Trigonella foenum-graecum. *Dependent variables included: estimated dietary energy intake; body composition; upper and lower body 1-RM strength, muscle endurance (80% of 1RM), anaerobic sprint power, and fasting clinical blood profiles (substrates, electrolytes, muscle and liver enzymes, red cells, white cells) and anabolic/catabolic hormones (free testosterone, cortisol, DHT, and estradiol) and metabolic hormones (insulin and leptin).

### Subjects

Forty nine resistance-trained (> 1 year) male subjects (Placebo: N = 23, 20 ± 1.9 years, 178 ± 6.3 cm, 85 ± 12.7 kg, 17 ± 5.6 %BF; Fenugreek: N = 26, 21 ± 2.8 years, 178 ± 6 cm, 90 ± 18.2 kg, 19.3 ± 8.4 %BF) participated in this study. Subjects were not allowed to participate in this study if they had any metabolic disorder including known electrolyte abnormalities; heart disease, arrhythmias, diabetes, thyroid disease, or hypogonadism; a history of hypertension, hepatorenal, musculoskeletal, autoimmune, or neurologic disease; if they were taking thyroid, hyperlipidemic, hypoglycemic, anti-hypertensive, or androgenic medications; and, if they had taken ergogenic levels of nutritional supplements that may affect muscle mass (e.g., creatine, HMB) or anabolic/catabolic hormone levels (androstenedione, DHEA, etc) within six months prior to the start of the study (table 1).

**Table 1:** Baseline characteristics of participants

Variable	Group: FEN	Group: PLA
**Age**	21.4 ± 2.8 yr	20.5 ± 1.9 yr
**Height**	178.1 ± 6.0 cm	178.5 ± 6.5 cm
**Weight**	90.2 ± 18.2 kg	85.7 ± 12.7 kg
**Body Fat %**	19.4 ± 8.4%	16.3 ± 4.8%

Abbreviations: FEN = fenugreek supplement group, PLA = placebo group

No significant differences (p > 0.05) between groups were observed.

Subjects were asked to maintain their normal dietary intake for the duration of the study and to refrain from ingesting any dietary supplement that contained potential ergogenic benefits. Subjects meeting eligibility criteria were informed of the requirements of the study and signed informed consent statements in compliance with the Human Subjects Guidelines of the University of Mary Hardin-Baylor and the American College of Sports Medicine.

### Entry and Familiarization Session

Subjects believed to meet eligibility criteria were then invited to attend an entry/familiarization session. During this session, subjects signed informed consent statements and completed personal and medical histories. Subjects meeting entry criteria were familiarized to the study protocol via a verbal and written explanation outlining the study design. This included describing the training program, familiarizing the subjects to the tests to be performed, and practicing the bench press, leg press, and Wingate.

### Testing Sessions

Following the familiarization/practice session, the subjects recorded all food and fluid intake on dietary record forms on four consecutive days preceding each experimental testing session in order to standardize nutritional intake. Dietary intake was assessed using the Food Processor Nutrition Software (ESHA, Salem, OR). Subjects were instructed to refrain from exercise for 48 hours and fast for 12-hours prior to baseline testing (T1). Subjects then reported to the Human Performance Lab for body composition and clinical assessments. Once reported to the lab, height was measured using standard anthropometry and total body weight was measured using a calibrated electronic scale (Health-o-meter^®^, Electromed Corp, Flint, MI) with a precision of +/-0.02 kg. Heart rate was determined by POLAR^® ^(Finland) heart rate monitor. Blood pressure was assessed in the supine position after resting for 5-min using a mercurial sphygmomanometer via standard procedures.

Subjects then had body composition determined using hydrodensitometry using standard procedures. Subjects reported to the Human Performance Lab in swimsuits and had their body weight determined out of water by an electronic scale. Body composition was analyzed using an EXERTECH (La Cresent, MN) body density measuring system that utilizes a weighing platform with electronic (load cell) weighing system connected to a PC. Calibration is conducted daily by establishing linear interpolation from 2 known weights. Data points were recorded with data acquisition software from the force transducer. Residual volume was estimated using standard procedures [18]. Subjects were submerged in warm water and asked to exhale a maximal amount of air while a signal from the force transducer produced a readable analog wave. The most stable waveform was selected, and the mean value was recorded. Subjects performed this procedure until at least 2 trials were within a 0.10% difference or a total of 7 trials were completed. Next, body density was calculated after weight was recorded in and out of water, and the Siri equation was used to calculate percentage of body fat [19]. Fat-free mass (FFM) was also calculated from the percentage of body fat [20].

Subjects then donated approximately 20 ml of fasting blood using venipuncture techniques of an antecubital vein in the forearm according to standard procedures. Blood samples were shipped to Quest Diagnostics (Dallas, TX) to run clinical chemistry profile, hepatic function, and whole blood cell counts. Blood samples were also centrifuged and aliquoted to microcentrifuge tubes and stored at -40°C for future analyses. Serum samples were then assayed in duplicate for the hormones free testosterone, Insulin, leptin, cortisol (Diagnostics Systems Laboratories, Webster, TX), and dihydrotestosterone (DHT), estradiol (Alpco Diagnostics, Windham, NH), using enzyme-linked immunoabsorbent assays (ELISA) and enzyme-immunoabsorbent assays (EIA) using a Wallac Victor-1420 microplate reader (Perkin-Elmer Life Sciences, Boston, MA), and the assays were performed at a wavelength or either 450 or 405 nm, respectively in the Exercise and Biochemical Nutrition Lab at Baylor University.

Subjects then performed 1 repetition maximum lifts (1-RM) on the isotonic bench press and leg press to assess strength and then muscular endurance. All strength/exercise tests were supervised by lab assistants experienced in conducting strength/anaerobic exercise tests using standard procedures. Subjects warmed-up (2 sets of 8 - 10 repetitions at approximately 50% of anticipated maximum) on the bench press. Subjects then performed successive 1-RM lifts starting at about 70% of anticipated 1-RM and increased by 5 - 10 lbs until the reaching a 1-RM. Subjects then rested for 10 minutes and warmed-up on the 45° leg press (2 sets of 8 - 10 repetitions at approximately 50% of anticipated maximum). Subjects then performed successive 1-RM lifts on the leg press starting at about 70% of anticipated 1-RM and increased by 10 - 25 lbs until reaching a 1-RM. Both 1-RM protocols were followed as outlined by the National Strength and Conditioning Association [21].

Following the strength assessments and 15 minutes of rest, subjects then perform a 30-second Wingate anaerobic capacity test using a Lode computerized cycle ergometer (*Groningen, Netherlands*). Cycle ergometer measurements (seat height, seat position, handle bar height, and handle bar position) were recorded and kept identical for each subject across testing sessions to ensure test to test reliability. Before leaving the lab, subjects were randomly assigned to a supplement group based on their body weight and given a training regimen. Subjects repeated all testing after 4 (T2) and 8 (T3) weeks of training and supplementation.

### Supplementation Protocol

Subjects were matched into one of two groups according to total body weight. Subjects were then randomly assigned to ingest in a double blind manner capsules containing 500 mg of a placebo (PL) or Fenugreek (Torabolic(tm) *Trigonella Foenum-Graecum) *(standardized for 70% TRIGIMANNOSE) (FEN) (Indus Biotech, India). The dosages investigated represent the current recommended dosages sold in nutritional supplements. Subjects ingested the assigned capsules once per day in the morning on non-training days and prior to their workout on training days for 8-weeks. The supplements were prepared in capsule form and packaged in generic bottles for double blind administration by Indus Biotech. Supplementation compliance was monitored by research assistants by watching them take the supplements prior to supervised workouts and by having the subjects return empty bottles of the supplement at the end of 4 and 8 weeks of supplementation. Subjects reported to a research assistant on a weekly basis throughout the study to answer a questionnaire regarding side effects and health status.

### Training Protocol

Subjects participated in a periodized 4-day per week resistance-training program, split into two upper and two lower extremity workouts per week, for a total of 8-weeks. This training regimen has shown to increase strength and lean body mass without additive dietary or supplementary interventions [22]. The subjects performed an upper body resistance-training program consisting of nine exercises (bench press, lat pull, shoulder press, seated rows, shoulder shrugs, chest flies, biceps curl, triceps press down, and abdominal curls) twice per week and a seven exercise lower extremity program (leg press, back extension, step ups, leg curls, leg extension, heel raises, and abdominal crunches) performed twice per week. Subjects performed 3 sets of 10 repetitions with as much weight as they can lift per set during weeks 1 thru 4 and performed 3 sets of 8 repetitions during weeks 5 thru 8, also with as much weight that could be lifted per set (typically 75-80% of 1RM). Rest periods between exercises lasted no longer than 3 minutes and rest between sets lasted no longer than 2 minutes. Training was conducted at the Mayborn Campus Center (MCC) at the University of Mary Hardin-Baylor under the supervision of trained research assistants, documented in training logs, and signed off to verify compliance and monitor progress. This training program has been shown to be a sufficient stimulus at inducing positive change in body composition and strength [22].

### Statistical Analysis

Separate 2×3 (treatment × time) repeated measure ANOVAs were used to assess all data. In circumstances where sphericity within groups could not be assumed due to large within group variances, the Hunyhs-Feldt epsilon correction factor was used to adjust within group F-ratios. For all significant group × time interactions and main effects, additional pair-wise comparisons were used to assess which time points yielded statistical significance between and within groups. Significance for all statistical analyses was determined using an alpha level of 0.05, and all data are presented as means ± standard deviations. All statistical procedures were analyzed using SPSS (Statistical Package for Social Science) version 16.0.

## Results

### Medical Monitoring, Dietary Analysis, and Training Volume

No subjects experienced any major clinical side effects related or unrelated to the study. However, several participants experienced gastrointestinal discomfort and/or mild stomach aches. All subjects completed the training protocol without any complications. Table 2 outlines all nutritional analyses data. No significant differences between groups (p > 0.05) were detected for total daily caloric intake, individual macronutrient intake, or training volume.

**Table 2:** Nutritional intake changes from baseline (T1) through week 8 (T3)

Variable	Group	Baseline (T1)	Week 4 (T2)	Week 8 (T3)	Between Group
**Total Calories**	FEN	2213 ± 926	2350 ± 799	2228 ± 986	G = 0.375
	PLA	2416 ± 916	2428 ± 850	3033 ± 1071	T = 0.323
					G × T = 0.214
**Carbohydrate (grams)**	FEN	266 ± 163	280 ± 111	262 ± 142	G = 0.937
	PLA	246 ± 110	245 ± 105	329 ± 176	T = 0.448
					G × T = 0.268
**Fat (grams)**	FEN	78 ± 40	82 ± 44	84 ± 55	G = 0.295
	PLA	91 ± 34	96 ± 41	118 ± 38	T = 0.277
					G × T = 0.505
**Protein (grams)**	FEN	116 ± 61	125 ± 57	105 ± 60	G = 0.772
	PLA	120 ± 50	116 ± 32	133 ± 41	T = 0.964
					G × T = 0.134

Abbreviations: FEN = fenugreek supplement group, PLA = placebo group

### Hematological Variables

There were no significant group × time interactions or main effects (p > 0.05) for red blood cell count, white blood cell count, triglycerides, cholesterol variables, liver enzymes or proteins, markers of kidney function or muscle damage.

### Body Composition

All body composition data are presented in table 3. Baseline total body weight was not significantly different (p = 0.326) between FEN and PL groups. There were no total body weight changes over the 8 week time course of the study between or within groups (p > 0.05). A significant main effect for time (p = 0.004) for lean body mass was observed, and further pair-wise comparisons revealed a significant increase in lean body mass for FEN at week 4 (p < 0.001) and week 8 (p < 0.001) compared with baseline. No such changes were seen in the PLA group (p > 0.005). A significant interaction effect (p < 0.001) and main effect for time (p < 0.001) occurred between groups for body fat percentage. Additional pair-wise comparisons displayed significant improvements in body fat percentage at week 4 (p < 0.001) and week 8 (p < 0.001) in FEN compared to baseline, while no such changes were noticed in PLA (p > 0.005).

**Table 3:** Body composition changes within and between groups

Variable	Group	Baseline (T1)	Week 4 (T2)	Week 8 (T3)	Between Group
**Body Weight**	FEN	90.2 ± 18.2	89.9 ± 18.2	90.4 ± 17.7	G = 0.305
**(kg)**	PLA	85.7 ± 12.7	85.0 ± 13.9	85.8 ± 12.4	T = 0.244
					G × T = 0.803
**Lean Mass**	FEN	157.7 ± 23.9	160.2 ± 23.8‡	162.6 ± 22.9‡	G = 0.640
**(kg)**	PLA	157.2 ± 19.5	156.4 ± 22.4	158.2 ± 19.5	T = 0.004†
					G × T = 0.057
**Body Fat %**	FEN	19.4 ± 8.4	17.8 ± 8.4 ‡	17.1 ± 8.6 ‡	G = 0.298
	PLA	16.3 ± 4.8	16.0 ± 4.8	15.9 ± 4.5	T < 0.001†
					G × T < 0.001†

Abbreviations: FEN = fenugreek supplement group, PLA = placebo group

Symbols: † = Significant between group difference (p < 0.05), ‡ = Within group difference from baseline (T1), p < 0.05

### Training Adaptations

Table 4 exhibits all training adaptation data. A significant group × time interaction (p = 0.008) and main effect for time (p < 0.001) was observed between FEN and PLA groups for bench press 1-RM, however pair-wise comparisons revealed no significant differences between FEN and PLA bench press 1-RM's at any time point. Pair-wise comparisons also showed significant increases in bench press 1-RM at week 4 (p < 0.001) and week 8 (p < 0.001) in comparison with baseline and from week 4 to week 8 (p = 0.002) in FEN. PLA experienced significant increases in bench press 1-RM at week 4 (p = 0.008) and week 8 (p = 0.004) when compared to baseline. A significant group × time interaction (p < 0.001) and main effect for time (p < 0.001) was observed between FEN and PLA groups for leg press 1-RM, as further pair-wise comparisons indicated a significant difference in FEN compared to PLA at week 8 (p = 0.019). Pair-wise comparisons also revealed significant increases in leg press 1-RM at week 4 (FEN: p < 0.001, PLA: p < 0.001) and week 8 (FEN: p < 0.001, PLA: p < 0.001) in comparison with baseline. No significant interactions or main effects (p > 0.005) were noted for muscular endurance repetitions on the bench press or leg press. A significant main effect for time (p = 0.002) was observed for wingate peak power, and further pair-wise comparison showed a significant increase in peak power for FEN at week 8 (p = 0.008). A significant interaction was detected for wingate mean power between FEN and PLA, but additional pair-wise comparison were unable to confirm any between or within group changes (p > 0.05).

**Table 4:** Training adaptations within/between groups from baseline (T1) through week 8 (T3)

Variable	Group	Baseline (T1)	Week 4 (T2)	Week 8 (T3)	Between Group
**Bench Press**	FEN	105 ± 26	111 ± 27‡	114 ± 27‡	G = 0.891
**1RM (kg)**	PLA	107 ± 22	109 ± 22‡	111 ± 22‡	T < 0.001†
					G × T = 0.008†
**Leg Press**	FEN	334 ± 74	384 ± 79‡	419 ± 87†‡	G = 0.077
**1RM (kg)**	PLA	316 ± 63	344 ± 66‡	364 ± 68‡	T < 0.001†
					G × T < 0.001†
**Bench Press**	FEN	7.9 ± 1.9	7.6 ± 1.9	8.2 ± 1.8	G = 0.091
**80% to failure**	PLA	7.3 ± 1.5	7.0 ± 1.5	7.5 ± 1.7	T = 0.154
					G × T = 0.984
**Leg Press**	FEN	12.2 ± 4.1	11.8 ± 3.8	10.8 ± 4.4	G = 0.836
**80% to failure**	PLA	12.0 ± 2.5	12.1 ± 2.8	11.3 ± 2.9	T = 0.168
					G × T = 0.821
**Peak Power**	FEN	1141 ± 222	1161 ± 198	1183 ± 200‡	G = 0.428
**(watts)**	PLA	1091 ± 215	1115 ± 231	1132 ± 237	T = 0.002†
					G × T = 0.974
**Mean Power**	FEN	628 ± 96	640 ± 107	643 ± 103	G = 0.363
**(watts)**	PLA	616 ± 90	609 ± 95	611 ± 85	T = 0.507
					G × T = 0.036†

Abbreviations: FEN = fenugreek supplement group, PLA = placebo group

Symbols: † = Significant between group difference (p < 0.05), ‡ = Within group difference from baseline (T1), p < 0.05, = Within group difference from week 4 (T2)

### Hormones

Hormonal data are presented in table 5. A significant group × time interaction effect over the eight week study period was detected for DHT concentrations, although pair-wise comparisons showed no between or within group changes (p > 0.05). A significant main effect for time was observed for leptin, however pair-wise comparions displayed no within group changes over time for FEN or PLA. A significant main effect for group was noticed for free testosterone, as further pair-wise analyses revealed significant differences between FEN and PLA at week 4 (p = 0.018) and week 8 (p = 0.027). No significant between or within group changes occurred for any other serum hormone variables (p > 0.05).

**Table 5:** Within and between group hormonal changes from baseline (T1) through week 8 (T3)

Variable	Group	Baseline (T1)	Week 4 (T2)	Week 8 (T3)	Between Group
**Estrogen**	FEN	102 ± 67	107 ± 55	109 ± 60	G = 0.196
**(pg/ml)**	PLA	83 ± 32	83 ± 31	91 ± 32	T = 0.173
					G × T = 0.563
**Cortisol**	FEN	75 ± 23	77 ± 27	74 ± 28	G = 0.805
**(mg/dl)**	PLA	88 ± 80	60 ± 21	85 ± 85	T = 0.418
					G × T = 0.324
**Insulin**	FEN	15 ± 8	13 ± 6	15 ± 8	G = 0.299
**(uIU/mL)**	PLA	15 ± 10	17 ± 10	16 ± 9	T = 0.962
					G × T = 0.060
**Leptin**	FEN	15 ± 14	13 ± 14	19 ± 16	G = 0.974
**(uIU/mL)**	PLA	14 ± 11	16 ± 12	17 ± 12	T = 0.044†
					G × T = 0.351
**Free**	FEN	40 ± 33	33 ± 22	36 ± 22	G = 0.020†
**Testosterone**	PLA	57 ± 47	66 ± 53†	67 ± 54†	T = 0.829
**(ng/ml)**					G × T = 0.318
**DHT (pg/ml)**	FEN	1263 ± 496	1152 ± 466	1144 ± 447	G = 0.921
	PLA	1187 ± 482	1156 ± 448	1258 ± 493	T = 0.134
					G × T = 0.033†

Abbreviations: FEN = fenugreek supplement group, PLA = placebo group

Symbols: † = Significant between group difference (p < 0.05)

## Discussion

The major findings of this study suggest that ingesting 500 mg of a commercially available botanical extract once per day for eight weeks in conjunction with a structured resistance training program can significantly impact body composition and strength in resistance trained males when compared to a placebo.

It is well documented that a controlled resistance training program can positively influence body composition across multiple populations [23-28]. The PLA group decreased body fat percentage over the 8 week period void of any experimental treatment however, this reduction was not found to be statistically significant. In contrast, the FEN group experienced a significant reduction in body fat percentage losing 2.34% compared to only 0.39% in the PL group. This change in body fat percentage is likely related to the significant increase in lean body mass observed exclusively in the FEN group. Together, these findings imply that supplementing with 500 mg of the commercially available supplement combined with resistance training can alter body composition to a greater extent than resistance training alone for 8 weeks. Woodgate and Conquer [29] investigated the effects of consuming a daily stimulant-free supplement containing glucomannan, chitosan, fenugreek, *G sylvestre*, and vitamin C in obese adults (age 20-50, BMI ≥ 30) while maintaining their normal dietary and exercise practices for six weeks. The experimental group significantly reduced their body fat percentage (-1.1% vs. 0.2%; p < 0.05) and absolute fat mass (-2.0 kg vs. 0.2 kg; p < 0.001) when compared with the placebo group. These results convey that the experimental proprietary blend significantly affected body composition more so than a placebo. The role that fenugreek alone played in altering body composition cannot be speculated, but in conjunction with glucomannan, chitosan, *G sylvestre*, and vitamin C, fenugreek did assist in the reported changes. Together, the present study and the findings of Woodgate and Conquer [29] demonstrate that fenugreek supplementation has the potential to improve body composition, specifically body fat percentage, over a chronic time period, although the mechanism of action has not been elucidated.

Strength increases resulting from a resistance training regimen are well established [24,30-35]. Initial strength changes occurring in untrained populations are attributable to neural adaptations [36,37], while individuals that have neurally adapted can experience hypertrophic changes that occur in a matter of weeks to months after the onset of resistance training [38]. In the present study, we employed an eight week, linear resistance training program that has established itself as an efficient stimulus for increasing muscular strength and lean muscle mass (hypertrophy) [22]. Over the course of eight weeks, the PL group significantly increased bench press (4.22%) and leg press (15.26%) 1-RM strength, indicating the resistance training program alone augmented upper- and lower-body maximal strength. The FEN group experienced a 9.19% increase in bench press 1-RM, but this increase was not influenced by the experimental treatment. In spite of this, the FEN group experienced an increases in bench press 1-RM from T1 to T2 and T2 to T3, while PLA only increased from T1 to T2. Based on this finding, it is possible that fenugreek can positively affect performance measures, such as those analyzed in the present study, over longer periods of time (8+ weeks). This hypothesis is also applicable to our Wingate peak power findings, as the FEN group underwent a significant increase from baseline at week 8. Significant differences were observed between FEN and PL groups at T3 for leg press 1-RM, as FEN underwent a 25.29% increase. No significant changes were observed for bench press or leg press muscular endurance tests or Wingate mean power. To our knowledge, there have been no investigations examining the effects of a dietary supplement containing fenugreek on muscular strength. However, one particular inquiry [39] evaluated the effects of two different dosings (10 mg/kg or 35 mg/kg) of galactomannan treatment, in comparison to testosterone treatment (10 mg/kg), on levator ani muscle weight in male castrated rats. At the end of six weeks, 35 mg/kg of galactomannan was as effective as the testosterone treatment at increasing the levator ani muscle and overall body weight in rats. An increase in a muscle's weight is reflective of muscle hypertrophy or an increase in the cross sectional area of muscle fibers. There is a direct relationship between a muscle's cross sectional area and overall strength of that particular muscle [40]. Therefore, if the levator ani muscle increased in cross sectional area, the possibility exists that a strength increase accompanied this adaptation, even though there were no strength measurements assessed in this study. The results from the present study suggest that 500 mg of a commercially available supplement can increase overall body strength during an 8 week period, or potentially over a more chronic time frame, in resistance trained males, and there is a possibility that a high dosage of a treatment (galactomannan) can increase muscle strength via muscle hypertrophy in rat models, even though no direct evidence subsists to support this claim.

Fenugreek supplementation is surrounded by assertions of having anabolic potential, even though there is no scientific data supporting this notion. In the present study we examined serum hormone variables that included free testosterone, DHT, estradiol, insulin, cortisol, and leptin over an eight week period. Of the above listed, no between or within group differences were observed for any of the measured hormone variables, except for free testosterone. Although a between group difference was noted for free testosterone at T2 and T3, it has limited relevance due to the fact that it did not significantly change over time. The investigation by Aswar and colleagues (2008) found no significant changes in serum testosterone levels in rats when treated with either a 10 mg/kg or 35 mg/kg dosage of galactomannan. This evidence coincides with our finding, which implies that the commercially available supplement lacks the potential for altering hormone values in combination with a resistance training regimen. Therefore, it is assumed that daily consumption of the 500 mg commercially available supplement in conjunction with a resistance training program has no anabolic effect on the hormonal status of resistance trained males.

## Conclusions

Based on the results of the study, we conclude that daily supplementation of 500 mg of the commercially available fenugreek supplement (Torabolic(tm)) in conjunction with an eight week, structured resistance training program can significantly increase upper- and lower-body strength, reduce body fat percentage, and thus improve overall body composition when compared to a placebo group under identical experimental protocols. The mechanisms responsible for these changes are not clearly understood due to the limited amount of research regarding fenugreek's potential for influencing anaerobic exercise performance and hormonal changes in animal as well as human populations. The commercially available supplement non-significantly impacted muscular endurance, hormonal concentrations and hematological variables. Future research might investigate different extractions and dosages of fenugreek on trained populations to determine if anabolic hormones can be altered and to ascertain if further strength and power output adaptations are possible that could ultimately enhance exercise performance.

## Competing interests

The authors declare that they have no competing interests.

## Authors' contributions

CW is the principal investigator. CP & BB assisted in data collection and coordinated the study. CP, CW, & LT analyzed data & wrote the manuscript. RK assisted in the grant preparation and securing grant funding. DW & LT analyzed blood variables. BC, LT, & CF consulted on study design, manuscript review and preparation. All authors have read and approved the final manuscript.
